# Trust of community health workers influences the acceptance of community-based maternal and child health services

**DOI:** 10.4102/phcfm.v9i1.1281

**Published:** 2017-05-29

**Authors:** Merridy Grant, Aurene Wilford, Lyn Haskins, Sifiso Phakathi, Ntokozo Mntambo, Christiane M. Horwood

**Affiliations:** 1 Centre for Rural Health, University of KwaZulu-Natal, South Africa; 2 School of Applied Human Science, University of KwaZulu-Natal, South Africa

## Abstract

**Background:**

Community health workers (CHWs) are a component of the health system in many countries, providing effective community-based services to mothers and infants. However, implementation of CHW programmes at scale has been challenging in many settings.

**Aim:**

To explore the acceptability of CHWs conducting household visits to mothers and infants during pregnancy and after delivery, from the perspective of community members, professional nurses and CHWs themselves.

**Setting:**

Primary health care clinics in five rural districts in KwaZulu-Natal, South Africa.

**Methods:**

A qualitative exploratory study was conducted where participants were purposively selected to participate in 19 focus group discussions based on their experience with CHWs or child rearing.

**Results:**

Poor confidentiality and trust emerged as key barriers to CHW acceptability in delivering maternal and child health services in the home. Most community members felt that CHWs could not be trusted because of their lack of professionalism and inability to maintain confidentiality. Familiarity and the complex relationships between household members and CHWs caused difficulties in developing and maintaining a relationship of trust, particularly in high HIV prevalence settings. Professional staff at the clinic were crucial in supporting the CHW’s role; if they appeared to question the CHW’s competency or trustworthiness, this seriously undermined CHW credibility in the eyes of the community.

**Conclusion:**

Understanding the complex contextual challenges faced by CHWs and community members can strengthen community-based interventions. CHWs require training, support and supervision to develop competencies navigating complex relationships within the community and the health system to provide effective care in communities.

## Introduction

Programmes involving community health workers (CHWs) are a component of many health systems, particularly in low- and middle-income countries. CHWs are broadly defined as members of a community, often chosen by the community and working within their own community, who are supported by the health system but have no professional training.^1^ Additionally, CHWs are usually volunteers but may receive a stipend.^2^ Using CHWs to deliver services to child carers in their communities has been shown to be effective in improving coverage of maternal, child and women’s health (MCH) interventions, leading to improvements in mortality.^1,3,4^ However, in some settings, high coverage of CHW visits and improvements in household knowledge and practice have not led to improvements in mortality.^5,6^ Implementing CHW programmes at scale has been a challenge, and it is crucial if CHWs are to function effectively that they be carefully selected, appropriately trained and receive adequate support.^7^

In South Africa, despite improvements in child and maternal mortality resulting from considerable investments in health services, particularly for HIV care and prevention of mother-to-child transmission (PMTCT), reducing deaths among mothers and children remains an important health challenge.^8,9^ An innovative strategy introduced by the South African Department of Health (DoH) to address this challenge is the re-engineering of primary health care (PHC).^9,10^ An important component of this initiative is the deployment of CHWs in communities to visit pregnant women and new mothers in their homes to provide key health promotion messages. The use of CHWs aims to provide appropriate, accessible care and bring care closer to mothers and babies, bridging the service delivery gap in underserved communities. CHWs can improve links between the community and the formal health sector, and thus improve retention in care, particularly in high HIV prevalence settings like South Africa, where PMTCT guidelines now recommend all HIV-infected pregnant women start lifelong antiretroviral treatment (ART).

Much of the success of any health intervention relies on positive and trusting relationships at individual, patient-provider and systemic levels.^11,12^ The magnitude of the role trust plays in health systems is often underestimated.^11,13^ At an individual level, successful uptake of services is largely determined by the relationship between the client and the health worker.^13,14^ In high HIV prevalence settings where patients risk stigma and loss of confidentiality, trust at a patient-provider level is essential if CHWs are to effectively support care in households and in the broader community. Furthermore, a lack of trust in the health system and between members of the health team also affects treatment compliance and health outcomes.^12^ At a systemic level, success of CHW interventions depends on high levels of community involvement and participation and a positive relationship between the CHW programme and the formal health system.^15^

Few studies have addressed and analysed the contextual challenges that impact on the successful functioning of CHWs.^16,17^ Understanding the challenges faced by CHWs and community members is important as it lays the foundation for successful CHW interventions.^17^ CHWs occupy a dual position as part of both the health system and the community, with a less well-defined role than that of formal health workers, so that an active process may be required to develop their roles and identities within the community.^18^ This study was conducted at a time when recent policy changes had led to the CHWs, role being extended to include provision of services for mothers and children in households. The study aimed to explore CHW acceptability and to identify possible barriers to successful implementation of this strategy. This article explores the importance of trust at all levels in the relationships between CHWs, the community and the formal health sector and how this affects service delivery.

## Research methods

### Study design

The study adopted an exploratory qualitative research design to access rich accounts of participants’ perceptions of CHW acceptability in the proposed community-based MCH intervention. A qualitative approach was most appropriate to explore the complexities, context or underlying themes of discourse,^19^ and focus group discussions (FGDs) were the most appropriate method to identify and explore the range of perceptions, attitudes and experiences of participants in relation to CHWs.

### Study setting

South Africa is a middle-income country with vast inequalities.^20^ This study was conducted in PHC clinics in five rural districts in KwaZulu-Natal (KZN) province, South Africa, between August and October 2012. KZN has the highest HIV prevalence in South Africa, with 37.4% of pregnant women attending government antenatal clinics testing HIV positive in 2012,^21^ and the highest infant and under-five mortality ratios in the country.^22^ KZN comprises 11 districts, of which 9 are rural. Health facilities are challenged by limited staffing, resources and infrastructure, and access to PHC clinics is affected by distance, financial constraints and transport availability. The main language spoken in the province is isiZulu.

There were approximately 10 600 CHWs employed by the South African DoH in KZN at the time of this study. CHWs received a two-week training to develop the skills to provide care and support to pregnant women, mothers, newborns and children in the community. CHWs were expected to provide appropriate health and nutrition education and implement simple, cost-effective interventions to identify and address common causes of illness and death among mothers and children.

### Study population and sampling

Participants were community members, professional nurses (PNs) and CHWs. Participants were purposively selected for inclusion in the FGDs based on their involvement with CHWs or child rearing. Eight clinics in five districts were selected to participate based on convenience and accessibility of CHWs. One PN from each clinic was purposively selected to participate for their knowledge and experience of working with CHWs. The PN then selected one CHW from the clinic catchment area.

Community members willing to participate in a discussion were purposively selected at clinics on the basis that they were the mother, father or grandmother of a child aged under 5 years, and therefore able to comment on the acceptability of CHW providing MCH services in the household (Table 1).

**TABLE 1:** Focus group participants by category and gender.

Type of participant	Number of groups	Participants	
		Male	Female
Community health workers	5	-	41
Professional nurses	5	-	37
Mothers (of children under 5 years)	3	-	25
Men (with children under 5 years in the household)	3	21	-
Grandmothers (of children under 5 years)	3	-	19
**Total**	**19**	**143**	

All participants received an information sheet explaining the purpose of the study and were given time to ask questions and provided written informed consent. All FGDs were digitally audio-recorded.

### Data collection and analysis

FGDs took place in a private room at the clinics and were facilitated by two trained interviewers. FGDs were convened according to the type of participant, with separate groups for CHWs, PNs and community members. Community groups were further separated into mothers, grandmothers and men. This was done to minimise the power dynamic that may arise from participants’ age, gender or professional status^23,24^ and to ensure that all voices were heard in the discussions.^25^ FGDs with CHWs and community members were conducted in isiZulu and those with PNs were conducted in English.

A semi-structured interview guide including scenarios or vignettes was used. Scenarios were used as a technique to explore the situational context of the proposed intervention, including sensitive issues like the disclosure of HIV status to the CHW, whether it was acceptable to visit a mother immediately after her baby’s birth or for a male CHW to visit at this time. Scenarios can allow participants greater control over the interaction by enabling them to determine at what stage, if at all, they introduce their own experiences to illuminate their abstract responses.^26^

**FIGURE 1:**
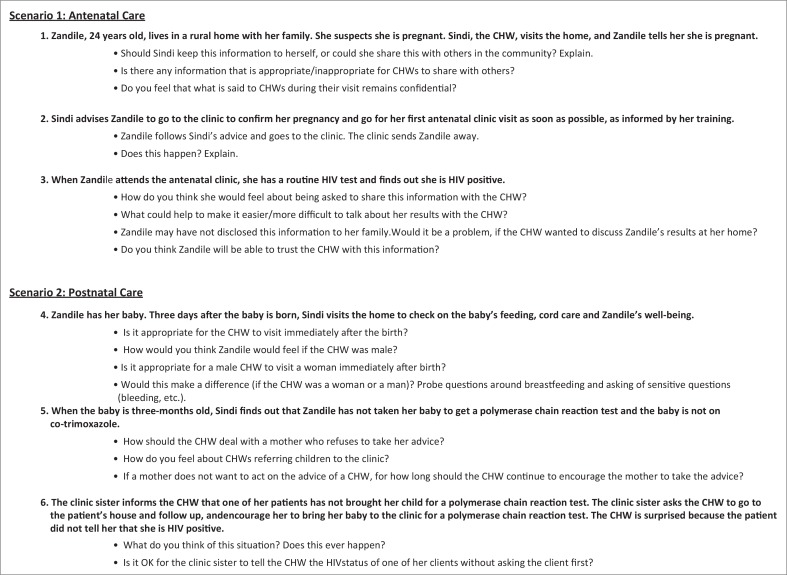
Example of scenarios used during focus group discussions.

Examples of scenarios used are shown in Figure 1. Issues around confidentiality, HIV disclosure, CHW gender and postnatal care were explored in all groups, with additional questions included specific to the category of participants being interviewed.

All FGDs were transcribed verbatim, translated into English where necessary, and coded by two experienced social scientists. An inductive approach was used and interpretive thematic coding was the primary analytic strategy.^24^ Transcripts were entered into qualitative data analysis software (NVivo version 10). After reading two transcripts, a codebook of themes was developed based on interview topics as well as new themes emerging from the data. This was repeated until all 19 transcripts had been reviewed and the codebook reached saturation where no new themes emerged. The analysis team worked together to resolve any interpretation discrepancies in the analysis process.

## Results

A total of 19 FGDs were conducted with 143 participants (Table 1).

Themes that emerged included the role of CHWs, the relationship of CHWs to the formal health sector, confidentiality and acceptability aspects of the intervention. The overarching theme of trust as an enabler and barrier for the acceptance of CHW services emerged within the household and community context. Perceptions of nurses in the health system regarding CHW service delivery also influenced CHW relationships with carers and the development of trusting relationships.

### Trust and confidentiality in the household

Lack of confidentiality and trust was expressed as a major barrier to CHW acceptability. CHW relations with child carers were complex, and successful interaction was based on trust. When CHWs and child carers were asked about their perceptions of CHWs and confidentiality, some felt that CHWs were trusted, while most felt that CHWs were not trusted by community members. However, there was agreement that CHWs who had a reputation for maintaining confidentiality would be accepted by the community.

‘It’s supposed to be like that [*confidential*], but we are not the same; in Zulu we say there are people that were kicked by a horse in the chest [*people who can’t keep secrets*]. Once a person gets home and they get excited and starts gossiping and say: “Hey I was at so and so’s and I found this and that”, you see. That is a crime. It is not supposed to happen. It is something that the CHW should keep to herself.’ (Community member, male, FGD)

PNs concurred, reporting that they frequently received complaints from members of the community regarding CHWs, lack of patient confidentiality, including disclosure of HIV status. ‘I know from experience that they (CHWs) do go around talking about other people’s problems.’ (Professional nurse, female, FGD)

It was reported that community members were reluctant to disclose sensitive information when they perceived that CHWs could not be trusted, and some chose to attend clinics in other areas to avoid being seen by local CHWs. CHWs sometimes breached ethical standards for confidentiality and were labelled as gossips. As members of the community, CHWs were familiar to the mothers and families they served, some of whom were their relatives or own friends: ‘Some people will not speak to the community health workers because they are relatives…. They think they will tell other people that they are ill.’ (Professional nurse, female, FGD)

One CHW acknowledged that sharing patient information with another CHW could lead to unintended disclosure of confidential information:

‘I think they [*CHWs*] do it by mistake, because we get told that each household’s information is confidential and should never be disclosed to a third party, but it happens that you find that so and so disclosed such and such information. When you and your colleague are close you talk and during your conversations you end up saying things and you tell her that I was at such and such a household. Such things happen.’ (Community health worker, female, FGD)

However, where relationships of trust did exist, they led to positive interactions between CHWs and mothers. There were community members who even asked CHWs to assist them in disclosing their HIV status to the family.

‘[*The CHW’s*] presence will help because she is the one who has encouraged her to go and make sure [*about her status*] at the clinic. The only problem would be fear of what they would say at home.’ (Community member, male, FGD)

CHW visits to households result in the CHWs interacting with other family members as well as with mothers. Community members felt that discussing confidential information at home was challenging if family members were present and could lead to unwanted disclosure of sensitive information. CHW visits sometimes caused contention or led to curiosity from family members, which undermined the trust relationship between the CHW, the client and the family. Particular complexities and safety around disclosure of HIV within the family were frequently raised during discussions:

‘It would be a problem if she has not told anyone [*her HIV status*] at home. When the health worker comes, she must state a reason for her visit and [*name*] can then look for a private place that they could go to so that they can talk alone, because she has not told anyone in her family about her status.’ (Community member, mother, FGD)

Community members suggested that CHWs should obtain client permission for household visits, and if women felt uncomfortable with a CHW visiting the household, another meeting place should be arranged. Some PNs expressed that CHWs could protect their patients and foster trust by suggesting to the family that the visit to the household is for a reason other than to discuss confidential issues, particularly regarding HIV:

‘If the patient has not disclosed to all the members then she [*CHW*] will make up a story. If she is a pregnant woman she will say she has come to check up on the baby’s health and things like that; they will make up a story.’ (Professional nurse, female, FGD)

Another factor influencing the development of a trusting relationship was the gender of the CHW, with women generally preferring female CHWs. Some participants wondered what a male CHW would be able to teach women: ‘Eish, what will he teach you?’ (Community member, mother, FGD). Some participants even felt that male CHWs were not to be trusted in terms of personal safety. ‘No one has ever told me that a woman has raped her child. It’s the males that are not trustworthy. They are the ones that rape our children.’ (Community member, mother, FGD)

However, several community members stated that as long as the CHWs were trained, gender did not matter. ‘If he has been trained in the same way, it makes no difference, because men deliver babies nowadays. They have been trained; there is no difference.’ (Community member, male, FGD)

The acceptability of male CHWs visiting households in the antenatal and postnatal periods was highlighted as a contentious issue by the male partners of women being visited and in the context of the sensitive and personal topics that might be discussed.

‘In terms of tradition, if someone is sick at home, we are from the rural areas; we are not from the townships. If a female person is sick at home, even I as the head of the household do not touch her. It’s the women who are neighbours who will come and assist her in whatever way that she needs to be assisted. We men will stay outside. We do not even go inside the house while the sick woman is being assisted by the other women. That is the traditional way.’ (Community member, male, FGD)

Nurses differed in their opinions on the issue of male CHWs. Some felt it was inappropriate to send male CHWs to a woman who had just delivered a baby, ‘No, it’s not proper … women are better … you will not send a male CHW to a delivered woman.’ (Professional nurse, female, FGD). However, one group of PNs cited how invaluable a particular male CHW was in their area.

‘I had a caregiver who was male who committed himself to help the sick people, even the old people; he is very good, he was diligent in his job, he reported to me every day, he was very good.’ (Professional nurse, female, FGD)

### Trust and the community

CHWs are recruited from within the community where they live, which means that they are likely to have both a personal and a service relationship with the people they visit. To explore how this was perceived by the participants, respondents were asked whether they would prefer CHWs to be recruited from their own community or from another community.

Participants were divided on the issue. Some participants said they were concerned about confidentiality and felt that a CHW from a different community would be more likely to keep information confidential:

‘I think they should come from a different community, not the same community as me. …Because if she is from the same community as me she may get tempted and end up telling other people [*about my secrets*].’ (Community member, male, FGD)

Others pointed to the importance of trust. They said that it was easier to develop a trusting relationship with someone who was familiar. Some community members felt that CHWs from their own community understood their context better and were more accessible and would therefore be more effective in their work.

‘ I think it is better if the health worker is from the same community as you, because she will know the lives of the people in your community. It will be easy for her to help the people because they are people from her community; a person cares about their community … rather than going to a community that they don’t know anything about.’ (Community member, mothers, FGD)

The CHWs themselves also had a range of views on the issue. Some thought it best to work in their own communities because they are known to the people they serve.

‘Yes it is nice to work in the community that you live in because you know each other. When you come by people won’t ask each other where you are coming from. They know you, even if you having conversations, they already know who you are.’ (Community health worker, female, FGD)

Those CHWs who preferred to work in other communities felt they would be more respected by outsiders.

‘Yes, I prefer working in a different community because they do not know me. In my community they might talk badly about me, but if they do not know me they will like and respect me and I will do the same to them.’ (Community health worker, female, FGD)

Another factor contributing to the development of trusting relationships between CHWs and community members related to the perceived competencies of CHWs as service providers. This played a vital role in helping community members feel confident and able to trust their ability to provide care. Trust in the CHWs’ abilities was related to their knowledge, training and skills, and competent CHWs made mothers feel their services could be trusted.

‘Once she [*client*] establishes that you have knowledge then trust is also established.’ (Community health worker, female, FGD)

At a community level, CHWs, ability to maintain client confidentiality came at a premium as they could suffer adverse consequences at the hands of the community if they were branded untrustworthy.

‘If they don’t keep confidentiality the clients will not trust them, they will not say anything to them, they will not be trusted. …They can also be sued … they can be chased away and beaten up.’ (Professional nurse, female, FGD)

### Trust and teamwork in the health system

CHWs and clinic staff are expected to work as a team to effectively promote optimal care for community members. PNs said that CHWs provide a link between communities and the health system to improve coverage of services.

‘They bridge the gap between the clinics and the community; they extend the health care from the facility to the community.’ (Professional nurse, female, FGD)

CHWs perceive their relationship with clinic staff differently. Some CHWs described a trusting relationship between PNs and CHWs, while others reported that referrals were not accepted by clinic staff, who undermined CHWs and showed a lack of confidence and trust in their ability to provide appropriate services.

‘ Even if we have those referral forms we still get undermined. They say we think we are doctors and they say this in front of the patient.’ (Community health worker, female, FGD);

CHWs felt their credibility was challenged by PNs, who made use of their help in busy times but treated them with contempt and disrespect when not needed. These power dynamics played out in the clinics and affected the perceived competency of CHWs by the community and undermined the trust individuals place in the CHW’s ability to provide care.

‘We feel as if we are not welcome. … If the clinic staff do not respect what we are doing there at the clinic, then how do they expect the community to respect us? They don’t value our presence.’ (Community health worker, female, FGD)

A number of nurses felt that formalising the CHW role in the health system would make CHWs more mindful of the issue of confidentiality, and most participants, including CHWs themselves, felt that CHWs required training in maintaining confidentiality.

‘CHWs should be educated. Training should be organised for them so that they can learn the kind of information that could be shared and which information cannot be shared.’ (Community health worker, female, FGD)

There were some respondents who believed training would make no difference as they felt that keeping information confidential was a personality factor.

‘They are taught well. They are given rules, but it’s just that they do not know how to keep secrets. If you can’t keep a secret, you can’t keep a secret.’ (Community member, grandmother, FGD)

## Discussion

Our data show that a lack of trust is a key contextual barrier to acceptance of community-based MCH services provided by CHWs in homes and suggest that successful community-based public health interventions are built on relationships of trust and good rapport with individuals, in the household and in the community. Furthermore, the perception that CHWs gossip was a barrier to trusting relationships at a systemic level with nurses and clinic staff and at an individual level with community members. Our study confirmed findings of other authors who state that community-based public health interventions are not merely about service delivery but also involve contextual factors and building relationships with community members,^11,13,17,23,27,28,29^ and that healthy collaboration at an individual level can improve outcomes.^30^ Trusting relationships also depend on CHW competencies in service delivery, which can be undermined by health care professionals’ perceptions of CHWs. Fostering relationships of trust between CHWs, community members and the personnel at facilities in the health system is vital in improving service delivery and uptake of services.

CHWs operate in a unique, complex environment, where interpersonal relationships test confidentiality. CHWs have to navigate interlinking relationships: being part of the community, being a neighbour, friend, as well as a service provider to families, and a supporter and collaborator with clinic staff. The complexity of this social environment challenges the development of the trusting relationships that are essential for uptake of health services. In this study, some participants felt local CHWs were more trustworthy, whereas others felt CHWs from another areas were better at keeping confidences. However, being a member of the community served is a cornerstone of the CHW model and is unlikely to change.

Additionally, the home, as a communal consultation space for receiving services, presents further challenges to privacy and confidentiality.^14^ The relationships of trust that CHWs need to establish with community members are played out in the household dynamics imposed on a very specific multifarious South African cultural context, which is further compounded by high HIV prevalence. The generally accepted CHW model of service delivery assumes that a community is a geographically defined space whose members share norms, values and status and that CHWs are on the same footing as their clients reducing the spatial and social distance that could hinder accessibility to homes.^23,27^ CHWs are assumed to be peers to their clients. In reality, socio structural factors, such as gender, age and social status, impact on CHWs’ ability to interact with all community members^17,23,27,29^ equally. This leads to social distance and restricting of information to certain members in the community, thus impacting the quality and effectiveness of the CHW–client intervention. Our data suggest that the gender of the CHW, in particular, impacted on relationships and influenced client confidences and trust. This finding is supported by other authors who have stated that age, gender and economic status of CHWs affected the way communities perceived them even before they began to perform their duties.^17,23,27,28,29^ CHW programmes should be sensitive to cultural norms regarding gender and, in some instances, the role of male CHWs in providing MCH services may have to be limited. Community leaders and other health team members can play a role in supporting the role of male CHWs in visiting mothers and children in their homes.

CHWs require skills and competencies to develop trust and rapport with other role players and recognise the role culture plays in their patient decision-making practices. CHWs are required to manage complex relationships in the household, community and clinic with minimal training and require appropriate training, support and supervision to do this effectively.

This study also suggested that trust was built from individual experiences and perceptions of the CHWs’ services. Thus, reports and accounts of others’ experiences and their own perceptions affected the degree of trust the community felt towards CHWs. Participants indicated that CHWs were more trustworthy if they were educated, knowledgeable and received adequate training. Thus, trust and training are interconnected.

Developing relationships of trust requires collaborative teamwork at a system level and depends on good communication between all members of the health team. Clinic staff play a key role in fostering confidence and trust among community members in the ability of CHWs to provide and link into appropriate services. Although some CHWs had good relationships with clinics, instances were described where the behaviour of the clinic staff undermined the credibility of CHWs. When clinic staff publicly undermine CHWs, this lack of respect and trust can strongly influence community perceptions of the CHW’s role.^11,14^ If nurses do not trust CHWs, this attitude may extend to the community, leading to CHWs’ competency being questioned and reducing acceptance of CHW services. The relationship with staff in the formal health sector has been shown to be a determinant of success in other CHW programmes.^15,30^ Thus, the relationship between clinic staff and CHWs needs to be actively strengthened and specific interventions designed to promote teamwork among the broader health team to include CHWs.

Trust issues are particularly challenging in high HIV prevalence settings where CHWs support HIV-infected mothers and their children. South Africa has recently introduced World Health Organization (WHO) Option B plus for PMTCT, which entails lifelong ART for all HIV-infected mothers from the time of HIV diagnosis.^31^ With this change, the role of CHWs becomes even more important as they are critical to supporting mothers and children on ART and improving retention in care. Trust of the health system and CHW competencies may be the most important determinants of effectiveness. CHWs cannot perform the required HIV-related services without collaboration with local clinics. Improvement in technical skills in the form of training and supervision will help build CHW competencies and in turn build trust in households and communities to improve links with the formal health system. Additionally, CHWs need personal and emotional skills in facilitating challenging consultations involving highly sensitive issues where confidences need to be kept.

It is widely acknowledged that effective supervision is crucial to the success of CHW programmes. Thus, upscaling CHW supervisors through training and support will provide them with the capacity to fulfil their supportive supervision and mentoring role more effectively. Supervision and support can improve motivation of CHWs and enhance their credibility in the eyes of the community. CHWs who function at an optimal level are capable of bringing timeous health care to vulnerable populations.

### Limitations

This was a qualitative study that was localised to a rural South African setting. All CHWs who participated in the focus groups were female. Inclusion of male CHWs may have added a different perspective to the results.

## Conclusion

The health system in South Africa is moving towards a more community-based PHC model with CHWs at its core. Our findings provide insight into the complex contextual challenges facing CHWs which may impact on their effectiveness. Failed trust relationships between CHWs and clinic staff creates a breach in the public health system and weakens the link between PHC and community. Specific attention needs to be placed on developing skills of CHWs to navigate the complex environment where they work, improving linkages and integration with the formal health sector and building teamwork at all levels.
